# The accuracy of preoperative ultrasound use in primary flexor tendon repair: A systematic review

**DOI:** 10.1016/j.jpra.2025.08.038

**Published:** 2025-09-17

**Authors:** Faderani Ryan, Seraj Shaikh Sanjid, Shahrjerdi Puneh, Kungwengwe Garikai, Kanapathy Muholan, Nikkhah Dariush, Mosahebi Afshin

**Affiliations:** a Department of Plastic Surgery, Royal Free Hospital, Pond St, London, NW3 2PS, United Kingdom; b Department of Surgery and Interventional Sciences, University College London, Royal Free Hospital, Pond St, London, NW3 2PS, United Kingdom; c Department of Plastic Surgery, John Radcliffe Hospital, Headley Way, Oxford, OX3 9DU, United Kingdom; d Department of Plastic Surgery, St Mary’s Hospital, Praed Street, London, W2 1NY, United Kingdom; e Department of Plastic Surgery, Charing Cross Hospital, Fulham Palace Rd, London, W6 8RF, United Kingdom

**Keywords:** Flexor tendon repair, Ultrasound, Diagnosis, Hand Surgery

## Abstract

**Introduction:**

Flexor tendon injuries disrupt hand function and require precise surgical repair. Traditionally assessed through physical examination alone, ultrasound scanning (USS) is emerging as a valuable tool, offering real-time, non-invasive imaging to assess injury type and extent. This review evaluates the USS's accuracy in preoperative flexor tendon assessment.

**Methods:**

Following PRISMA guidelines, a systematic review was conducted using MEDLINE, EMBASE, and Web of Science. Studies on primary flexor tendon injuries assessed with preoperative USS were included, while non-clinical studies, animal models, and incomplete datasets were excluded. Two reviewers screened articles, with disagreements resolved by a third reviewer.

**Results:**

From 1354 initial studies, nine met inclusion criteria, comprising one RCT, four prospective, and four retrospective studies. In total, 450 patients (593 tendons) with diverse injuries were assessed. USS sensitivity and specificity were high for complete tears (88.78 % and 92.23 %) but lower for partial tears (0.67 % and 66 %).

**Discussion:**

USS provides an effective, non-invasive method for identifying complete flexor tendon injuries, reducing the need for exploratory surgery, facilitating targeted incisions, and potentially shortening operative times. By enabling accurate surgical planning, USS helps minimize dissection and trauma, aligning with sustainable healthcare practices. Its application in delayed presentations also guides repair strategies, enhancing outcomes through more precise tendon localization.

**Level of evidence:**

IIIA.

## Introduction

Flexor tendon injuries, if left untreated, can lead to significant hand deformity and disability, often necessitating surgical repair to restore hand function.^1^ Such injuries, commonly caused by lacerations, occupational or sports accidents, disrupt tendon anatomy and function, requiring preoperative assessments to guide both operative and non-operative management.^2^

Physical examination has been the traditional gold standard for pre-operative assessment of flexor tendon injuries. Increasingly, ultrasound scanning (USS) has emerged as an adjunct to physical examination of flexor tendon injuries, offering real-time, high-resolution images.^3^ USS is non-invasive, inexpensive, widely available, and radiation-free. Studies have shown various applications of USS regarding flexor tendon repairs. For example, USS has been utilized to evaluate flexor tendon integrity and distinguish between partial and complete tears.^4^, ^5^, ^6^ The ability of USS to visualize soft tissue structures, measure tendon retraction, and assess surrounding anatomical features makes it a valuable tool in the preoperative planning for flexor tendon repairs.^7^, ^8^, ^9^

This review seeks to determine the overall utility of USS in preoperative assessment for flexor tendon repairs. Preoperative evaluation with USS could lead to better surgical outcomes, reduced operative times, minimized complications, and improved patient-reported outcomes and satisfaction. A review of the literature suggests that no prior systematic reviews have addressed the use of preoperative ultrasound in the surgical repair of flexor tendon injuries.

### Aims

How accurate is USS of flexor tendons in identifying primary flexor tendon injuries in the pre-operative phase?

## Methods

This systematic review was conducted and reported according to the Preferred Reporting Items for Systematic Reviews (PRISMA) guidelines.^10^

### Search strategies

A systematic review of the literature was performed using the MEDLINE (OvidSP), EMBASE (OvidSP) and WoS (Web of Science) databases. The search strategy included a combination of text words and Medical Subject Headings (MeSH) terms. The following search strategy was tailored across the three databases to ensure optimal search results:

((TS=(Flexor tendon* OR flexor digitorum superficialis adj5 tendon* OR FDS adj5 tendon* OR flexor digitorum profundus adj5 tendon* OR FDP adj5 tendon* OR flexor policis longus adj5 tendon* OR FPL adj5 tendon* OR Flexor zone*)) AND TS=(injur* OR ruptur* OR lacerat* OR sever* OR avuls* OR tear*)) AND TS=(Repair* OR Reconstruct* OR surg*)) AND TS=(finger* OR hand* OR hand injur* OR finger injur*)) AND TS=(ultrasound* OR ultrasonography* OR sonograph* OR echograph).

No language or publication restrictions were applied.

### Inclusion criteria

All primary clinical studies involving use of pre-operative ultrasound imaging in primary flexor tendon sheath repairs involving all patients were included.

### Exclusion criteria

The exclusion criteria were studies that reported no clinical data pertaining to ultrasound imaging in flexor tendon sheath repairs, post-operative ultrasound imaging, non-English language articles, *in vitro* studies, all review articles and studies in animal or cadaveric models. Abstracts, conference proceedings without full text, and ongoing trials with incomplete data sets were excluded.

### Outcome measures

The primary outcome measures were number of tendon assessed by USS, USS device, mechanism of injury, number of complete and partial injuries confirmed by USS.

### Study selection and data management

Study selection was conducted in a two-stage process. Two reviewers initially screened titles and abstracts for potential eligibility after excluding duplicate studies. Next, studies identified as relevant underwent full-text review by both reviewers. Discussion and referral to a third reviewer were used to resolve conflicts between the reviewers in the study selection. The data from all full-text articles accepted for final analysis were independently retrieved using a standardized data extraction form. Any discrepancies between reviewers at this point were resolved through discussion or referral to senior author. The search results were recorded including abstracts, full-text articles, and records of reviewers’ decisions, including reasons for exclusion.

The extracted data includes details on study characteristics, patient demography, the type of ultrasound used, details regarding the flexor tendon injury, and patient-reported outcome measures. Data was extracted from the studies as presented or calculated.

## Results

### Search results

**Figure 1:**
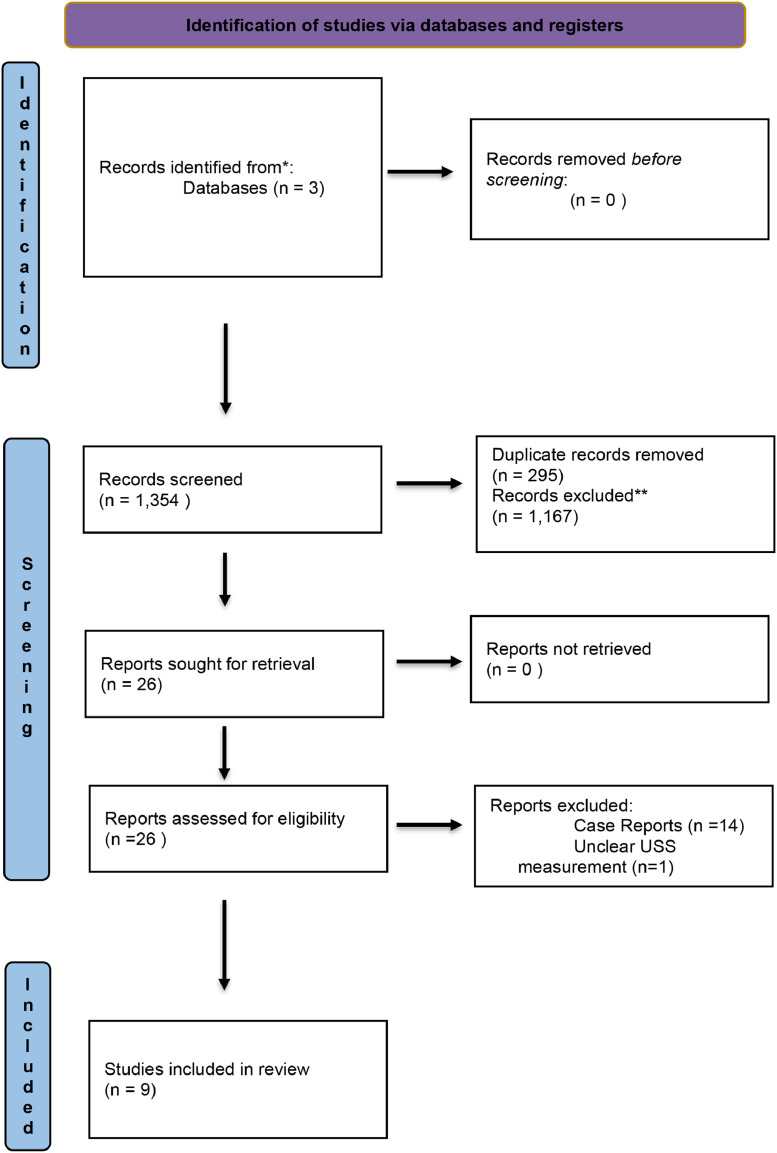
PRISMA diagram.

An initial 1354 studies were found from the initial searches. Initial searches were conducted on MEDLINE (OvidSP), EMBASE (OvidSP) and WoS (Web of Science) databases. 295 duplicate papers were identified and removed. The screening process and conclusion of finalized articles are illustrated in the PRISMA diagram (Figure 1). 26 papers were then examined for eligibility and full-paper review. Following a full-paper review, 17 publications were excluded. These excluded studies were either case reports or did not differentiate the time of USS. Following exclusion, nine articles were included for final analysis.

### Study characteristics

There was one randomized control trial, four prospective cohort studies, and four retrospective case-control studies. All studies used USS in the preoperative phase.

### Study demographics

Across the nine papers, 593 flexor tendons were identified in 450 patients: men (*n* = 296) and women (*n* = 154). Traumatic flexor tendon injuries resulted from street fights, workplace injuries, gunshots, saw blades, knives, tin cans, band saws, barbecue grills or self-inflicted wounds (Table 1, Table 2, Table 3, Table 4).

**Table 1:** Summary of included studies.

Study	Mechanism/Injury type	No. of tendons (*n* =)	Type of USS	Complete tears (*n* =)	Incomplete tears (*n* =)	No tear (*n* =)
Wang et al., 1999^37^	Trauma (unspecified)	8	AT: HD1–3000 5–9 MHz hockey stick linear probe	3	Not recorded	5
Lee et al., 2000^6^	Gunshot, saw blade, knife, tin can, band saw, barbecue grill	18	Advanced technology laboratories ultramark 9 ultrasound scanner (Bothell, WA), with a 38-mm L10–5 MHz linear transducer	6	1	12
Jeyapalan et al., 2008^9^	Trauma (unspecified)	13	Siemens Antares (Siemens AG, Erlangen, Germany) ATL HDI 5000 (ATL ultrasound, Philips medical systems company, Bothell, USA).	1	0	2
Zhang et al., 2012^8^	Work accidents, self-inflicted	185	LOGIQ 9, GE Healthcare, 10 MHz	79	16	0
Al-Hourani et al., 2018^38^	Trauma (unspecified)	9	Philips 15 MHz iU22 diagnostic US system with L15–7IO transducer	8	Not recorded	1
Akhavan et al., 2019^39^	Street fight: 61.6 %, work: 25 %, home: 13.4 %	214	Bedside “point-of-care” USS 12 MHz SonositeTM surface probe.	48 (inc incomplete)	48 (includes complete)	165
Bekhet et al., 2021^5^	Injured site: first digit (17.5 % *n* = 7), second digit (20.0 % *n* = 8), third digit (15.0 % *n* = 6), fourth digit (15.0 % *n* = 6), fifth digit (15.0 % *n* = 6), wrist (7.5 % *n* = 3), palm (10.0 % *n* = 4). Injured zone: I (10.0 % *n* = 4), II (47.5 % *n* = 19), III (12.5 % *n* = 13), IV (5.0 % *n* = 2)	50	LOGIQ P9 ultrasound system from GE Healthcare, Chicago, IL,USA) with Doppler	21	9	5
Meisami et al., 2021^40^	Trauma (unspecified)	80	Toshiba device	52	25	3
Bezirgan et al., 2023^41^	Zone 1 (*n* = 6), Zone 2 (*n* = 14), Zone 5 (*n* = 1)	21	Not Recorded	Not recorded	Not recorded	Not recorded

**Table 2:** Summary of tendon injuries.

Study	No. of tendons	Injured tendons	Flexor zone of injury	Open/Closed
Wang et al., 1999^37^	20	9/20 FDP 7/20 FDS 4/20 FPL	Not specified	19/20 Open 1/20 Closed (#)
Lee et al., 2000^6^	17	8/17 FDP 7/17 FDP + FDS 2/17 FPL	3/17 Zone 1 6/17 Zone 2 1/17 Zone 3 10/17 investigated post tendon repair	3/17 Closed 14/17 Open
Jeyapalan et al., 2008^9^	185	185 FDP + FDS	100 % Zone 2	Not specified
Zhang et al., 2012^8^	9	3 FDP + FDS 1 FDS + A2/A3 pulley 2 FDP 2 FPL	Not specified	Not specified
Al-Hourani et al., 2018^38^	214	Not specified	Not specified	214 Open (penetrating injury)
Akhavan et al., 2019^39^	50	14/50 FDS 26/50 FDP 1/50 FCU 7/50 FPL 1/50 FCR 1/50 PL	4/40 Zone I 19/40 Zone II 5/40 Zone III 2/40 Zone IV 2/40 Zone V 1/40 T1 6/40 T2 1/40 T3	22/35 Open 13/35 Closed
Bekhet et al., 2021^5^	80	1.8 % FCR + FCU 13.3 % FDP 21.2 % FDS	Not specified	80 Open (penetrating hand trauma)
Meisami et al., 2021^40^	21	Not specified	6/21 Zone 1 14/21 Zone 2 1/21 Zone 5	Not specified
Bezirgan et al., 2023^41^	20	9/20 FDP 7/20 FDS 4/20 FPL	Not specified	19/20 Open 1/20 Closed (#)

FPL, Flexor pollicis longus; FDP, flexor digitorum profundus; FDS, flexor digitorum superficialis; FCR, flexor carpii radialis; FCU, flexor carpii ulnaris; A pulley, annular pulley.

**Table 3:** Summary of accuracy USS results (complete tears).

Study	Sensitivity (%) ± (CI)	Specificity (%) ± (CI)	PPV (%)	NPV (%)	LR+	LR-	Diagnostic Odds Ratio (DOR)
Wang et al., 1999^37^	100 (100, 100)	100 (100, 100)	100	100	N/A	N/A	N/A
Lee et al., 2000^6^	87.5 (61, 100)	91.7 (69.6, 100)	87.5	91.67	10.5	0.1363	77.035
Jeyapalan et al., 2008^9^	100 (100, 100)	100 (100, 100)	100	100	N/A	N/A	N/A
Zhang et al., 2012^8^	100 (100, 100)	100 (100, 100)	100	100	N/A	N/A	N/A
Al-Hourani et al., 2018^38^	75 (45, 100)	99.8 (96.7, 100)	99.99	1	301.375	0.251	1200.7
Akhavan et al., 2019^39^	100 (100, 100)	99.4 (97.2, 100)	97.99	100	166.667	0	N/A
Bekhet et al., 2021^5^	100 (100, 100)	100 (100, 100)	100	100	N/A	N/A	N/A
Meisami et al., 2021^40^	86.5 (77.2, 95.8)	72.2 (60, 84.4)	81.8	78.9	3.12	0.187	16.684
Bezirgan et al., 2023^41^	50 (28.6, 71.4)	67 (46.9, 87.1)	86	25	1.515	0.7463	2.03

CI, Confidence interval; PPV, positive predictive value; NPV, negative predictive value; LR+, positive likelihood ratio; LR-, negative likelihood ratio.

**Table 4:** Summary of accuracy USS results (incomplete tears).

Study	Sensitivity (%) ± (CI)	Specificity (%) ± (CI)	PPV (%)	NPV (%)	LR+	LR-	Diagnostic Odds Ratio (DOR)
Wang et al., 1999^37^	No documentation on partial tears						
Lee et al., 2000^6^	0	0	0	0	0	0	N/A
Jeyapalan et al., 2008^9^	No documentation on partial tears						
Zhang et al., 2012^8^	100 (100, 100)	100 (100, 100)	100	100	N/A	N/A	N/A
Al-Hourani et al., 2018^38^	No documentation on partial tears						
Akhavan et al., 2019^39^	No documentation on partial tears						
Bekhet et al., 2021^5^	100 (100, 100)	100 (100, 100)	100	100	N/A	N/A	N/A
Meisami et al., 2021^40^	86.5 (73.1, 99.9)	72.2 (54.6, 89.8)	81.8	78.9	3.12	0.187	16.684
Bezirgan et al., 2023^41^	No documentation on partial tears						

CI, Confidence interval; PPV, positive predictive value; NPV, negative predictive value; LR+, positive likelihood ratio; LR-, negative likelihood ratio.

### Quality of included studies

The quality of included studies was assessed using the GRADE tool.^11^ Limitations, results inconsistency, evidence indirectness, imprecision, and publication bias were all considered when assessing each study. Three studies were deemed low quality, one study was assessed as moderate quality, and four studies were high quality (see Table 5). Studies were generally considered low/ moderate quality due to lower effect magnitudes, inconsistency, and indirectness.

**Table 5:** Study design and GRADE rating.

Study	Study design	Quality of assessment (GRADE)
Wang et al., 1999^37^	Retrospective cohort study	Low
Lee et al., 2000^6^	Prospective cohort study	Low
Jeyapalan et al., 2008^9^	Retrospective case control study	Low
Zhang et al., 2012^8^	Randomized controlled study	High
Al-Hourani et al., 2018^38^	Retrospective case control study	Moderate
Akhavan et al., 2019^39^	Prospective cohort blind study	High
Bekhet et al., 2021^5^	Prospective cohort study	High
Meisami et al., 2021^40^	Prospective cohort study	High
Bezirgan et al., 2023^41^	Retrospective case control study	High

### Risk of bias

The only randomized control trial had a high risk of bias due to allocation concealment bias. ROB2 was used to assess RCTs in this study^12^ (see Table 6). Included non-randomized clinical studies were assessed against the ROBINS-I risk of bias tool.^13^ Non-RCTs generally had a moderate to high risk of bias due to selection, comparability and outcome metrics (see Table 7).

**Table 6:** ROB 2 risk of bias analysis of RCTs.

**Table 7:** ROBINS-I risk of bias analysis of Non-RCTs.

### Outcomes

Outcomes were assessed by calculating the overall positive predictive value (PPV), negative predictive value (NPV), positive likelihood ratio (LR+), negative likelihood ratio (LR-) and diagnostic odds ratio (DOR). Overall pre-operative USS findings for complete primary flexor tendon injuries were as follows (pooled mean ± SD): sensitivity (88.78 % ± 17.06), specificity (92.23 % ± 13.17), PPV (94.81 % ± 7.46), NPV (77.40 % ± 37.65), LR+ (96.64 ± 134.20), LR- (0.26 ± 0.28) and DOR (324.11 ± 585.29). Summary of pre-operative USS findings for incomplete primary flexor tendon injuries were as follows (pooled mean ± SD): sensitivity (0.67 % ± 0.58), specificity (66 % ± 0.57), PPV (63 % ± 0.55), NPV (67 % ± 0.58), LR+ (19.23± 27.19), LR- (0 ± 0) and DOR (N/A).

## Discussion

This systematic review has shown that USS is capable of predicting flexor tendon injuries in both pre- and peri‑operative settings following trauma. USS identified 236 complete and partial flexor tendon injuries amongst the 593 injured tendons. This systematic review demonstrated high sensitivity and specificity in identifying complete primary flexor tendon injuries using USS.

### Benefits of USS use

USS provides considerable advantages in the assessment and diagnosis of flexor tendon injuries during the pre-operative phase. USS provides real-time, dynamic imaging, allowing clinicians to assess tendon integrity and function without requiring invasive diagnostic procedures.^5^^,^^7^ This eliminates the risks associated with exploratory surgery, which is often used to determine the extent of tendon damage. Avoiding this using USS can potentially prevent unnecessary surgical interventions for patient care.^14^ Across the medical literature, USS has proven to significantly reduce the time, cost, and environmental impact of operative procedures.^15^ The materials used intraoperatively and the energy consumption required for operating rooms add to the environmental footprint and medical waste.^16^ Avoiding surgery through precise USS diagnosis could reduce these environmental impacts.

USS is valuable for visualizing flexor tendons^17^ in real-time, thereby avoiding complications such as neurovascular injury. In clinically indicated tendon explorations, USS assists in targeted and tailored incisions, minimizing extensive tissue dissection and reducing operative time.^18^ This, in turn, could decrease patient recovery time, thereby lowering healthcare costs and freeing up surgical resources.^19^ The use of USS could potentially lead to a reduction in treatment delays, improved patient flow pathways, and enhanced clinical decision-making in trauma settings.

Localizing the proximal cut-end of the tendon in flexor tendon injuries through USS is beneficial both in the initial operation and in delayed presentations.^6^ With these levels of specificity and sensitivity, USS could help in tailored surgical management by determining suitability for primary repair. In acute cases, real-time tendon visualization can better facilitate minimally invasive techniques, such as wide-awake local anesthesia with no tourniquet (WALANT).^20^ Using USS in conjunction with WALANT could facilitate higher precision in repairs and avoid extensive tissue dissection, thus resulting in less intraoperative trauma and faster recovery times.^21^

Minimizing scarring is crucial, as extensive scarring in flexor tendon repairs is associated with higher rates of adhesions.^22^ Adhesions can limit tendon gliding, worsen stiffness, reduce range of motion, and exacerbate chronic pain. Furthermore, prolonged healing times due to scarring can significantly impact a patient’s quality of life and daily activities.^23^ Complications increase with larger incisions, such as a higher risk of wound breakdown and infection, requiring more dressings, antibiotics, frequent outpatient appointments, and potentially extended hospital inpatient stays.

A minimally invasive approach that encourages early mobilization may reduce these aforementioned risks.^24^ Fewer post-operative complications and faster rehabilitation may prevent further revision operations and intensive physiotherapy, thereby improving long-term outcomes and alleviating the burden on hand trauma units and other healthcare costs.^25^

Overall, USS offers a non-invasive, potentially cost-effective, and environmentally-friendly alternative to traditional diagnostic and surgical approaches for flexor tendon injuries, enhancing patient care while minimizing unnecessary resource use.

### User operability

Across the analyzed studies in this systematic review, USS operators varied from experienced surgeons to experienced emergency clinicians, radiologists, and radiographers. The operator's experience heavily influences the effectiveness of USS in diagnosing and managing flexor tendon injuries.^26^ Clinicians with significant USS experience are more adept at identifying nuanced tendon pathology, such as tears, inflammation, and vascularity, demonstrating a clear advantage in preoperative and perioperative settings, which allows for better preoperative planning and perioperative guidance.^7^^,^^27^ USS requires a strong understanding of hand anatomy and the ability to interpret dynamic imaging in real-time, which can prove challenging for novices.^28^ Several countries have established training courses and assessments in USS to lessen the learning curve for trainee clinicians and medical students.^29^, ^30^, ^31^

Despite this learning curve, USS is relatively user-friendly once proficiency is attained. Portable machines and technological advancements have made USS accessible and practical, even in peri‑operative settings.^7^ With the development of pocket ultrasound devices such as Butterly iQ™^32^ and Clarius,^33^ trained surgeons can readily perform perioperative and intraoperative scans, eliminating the need for referral to sonographers.^34^ For surgeons, using USS can streamline decision-making, potentially reducing the need for more invasive diagnostic techniques. Training programs and standardized protocols can help bridge the gap between novice and experienced users, promoting a broader user base for flexor tendon injury management.^35^

### Partial flexor tendon injuries

Determining the degree of partial tendon injury is necessary for clinical decisions to operate; typically, a partial flexor tendon injury of >60 % cross-sectional area should be repaired.^36^ However, following the main findings of this systematic review study, USS has been deemed ineffective in detecting partial flexor tendon injuries of the hand. Poor sensitivity, specificity, PPV, and NPV values demonstrate the ineffective use of USS in such cases. Across the finalized studies, the degree to which a partial injury was elicited on USS was not recorded by the majority of studies (*n* = 8).

Despite demonstrating the high sensitivity and specificity of determining complete primary flexor tendon injuries, this review has not been able to demonstrate efficacy and accuracy in partial injuries. Across the studies examined, there were instances of under-reporting of partial tendon injuries or amalgamating partial and complete injuries when discussing accuracy statistics. In such cases, this systematic review has appropriately recorded such events for clarity. Given the results of this systematic review, the use of USS is recommended to identify complete flexor tendon injuries.

In conclusion, there are currently no systematic reviews in the literature that have assessed the accuracy of USS in evaluating flexor tendon injuries during the pre-operative phase. Only one RCT has focused on this subject,^8^ and a small sample size limits its findings. The findings of this systematic review demonstrate the accuracy and utility of USS in diagnosing and monitoring complete flexor tendon injuries, supporting its integration into standard clinical practice. More high-quality RCTs with larger cohorts are necessary to validate these results further and reinforce the role of USS in managing flexor tendon injuries.

## Contributions

**Ryan Faderani:** Conceptualisation, data curation, writing and editing. **Shaikh Sanjid Seraj:** Data curation, formal analysis, writing. **Puneh Shahrjerdi:** Data curation, formal analysis, writing. **Garikai Kungwengwe:** Data curation, formal analysis. **Muholan Kanapathy:** Review and editing. **Dariush Nikkhah:** Review and editing. **Afshin Mosahebi:** Review and editing.

## Funding

None.

## Ethical approval

Not required.

## Conflicts of interest

None declared.
